# Neural and Kinematic Characteristics of Reaching in Autistic Children During Movement Observation, Execution, and Synchronization: An fNIRS Study

**DOI:** 10.3390/brainsci16050540

**Published:** 2026-05-20

**Authors:** Wan-Chun Su, Daisuke Tsuzuki, Anjana Bhat

**Affiliations:** 1 School of Kinesiology, Louisiana State University, Baton Rouge, LA 70802, USA; wanchunsu@lsu.edu; 2 Department of Information Science, Faculty of Science and Technology, Kochi University, Kochi 780-8520, Japan; 3 Department of Physical Therapy, University of Delaware, Newark, DE 19713, USA; 4 Biomechanics & Movement Science Program, University of Delaware, Newark, DE 19713, USA; 5 Department of Psychological & Brain Sciences, University of Delaware, Newark, DE 19716, USA

**Keywords:** autism, neural activity, movement kinematics, interpersonal synchrony, functional near-infrared spectroscopy (fNIRS), inertial measurement unit

## Abstract

**Highlights:**

**What are the main findings?**

- Autistic children exhibited atypical reaching kinematics indicative of poor feedforward control and greater reliance on feedback control during reaching.
- In terms of cortical activation, autistic children showed reduced activation in the prefrontal and temporal cortices along with increased frontoparietal activation, indicating greater cognitive involvement during motor tasks.

**What are the implications of the main findings?**

- These behavioral and cortical activation patterns may serve as potential biomarkers for screening of autistic children after confirming similar findings in younger autistic children.
- The compensatory patterns observed in this study’s autistic participants can inform intervention development aimed at improving social-motor performance of autistic children.

**Abstract:**

Background/Objectives: Children with Autism Spectrum Disorder (ASD, here on termed autistic children) exhibit motor difficulties in social and non-social contexts. Although previous studies have reported behavioral and neural characteristics, their relationship remains largely unexplored. The current study aimed to investigate the behavioral and neural mechanisms underlying interpersonal synchrony in autistic children using simultaneous kinematic and Functional Near-Infrared Spectroscopy (fNIRS) recordings. Methods: Fifty-eight autistic or non-autistic children participated (mean age = 10.1, standard error = 0.3). fNIRS and an inertial measurement unit were used simultaneously to record the neural activity over frontotemporal and parietal regions and arm movement kinematics during a reach-to-clean-up task across three conditions: Watch—the child observed the tester clean up the blocks; Do—the child cleaned up the blocks independently; and Together—the child and tester cleaned up the blocks synchronously. Results: Behaviorally, autistic children demonstrated longer movement displacement, higher average velocity and acceleration, and a greater number of movement units. In terms of cortical activation, autistic children showed hypoactivation in the bilateral precentral gyrus and right inferior parietal lobe, along with hyperactivation in the right middle frontal gyrus, left inferior frontal gyrus, and left inferior parietal lobule. Correlations between kinematic and neural measures suggest that autistic children rely more on online/feedback control to compensate for reduced feedforward control. Conclusions: This study reveals unique compensatory strategies in autistic children, highlighting the connections between neural and behavioral characteristics. These findings have strong potential to inform the development of ASD screening tools and to guide targeted intervention strategies.

## 1. Introduction

Autism Spectrum Disorder (ASD) is a prevalent neurodevelopmental condition, affecting approximately 1 in 31 children in the United States [1]. Although the core diagnostic features include impairments in social communication and the presence of restricted and repetitive behaviors [2], a substantial proportion of autistic children also exhibit motor difficulties [3,4,5]. These difficulties affect children’s gross (e.g., ball skills, postural control) and fine motor skills (e.g., handwriting) and significantly impact their participation in daily activities [6,7,8]. Motor challenges are often exacerbated during tasks with social components, such as imitation, interpersonal synchrony, and cooperative/competitive joint actions, collectively referred to as social motor tasks [9,10,11,12]. Converging evidence further indicates atypical neural activation in frontal, temporal, and parietal regions are associated with social motor difficulties in autistic children [13,14,15,16,17]. A better understanding of neural mechanisms may help elucidate how motor impairments interact with the core social communication impairments in autistic children [18,19,20]. In the current study, we simultaneously recorded Functional Near-Infrared Spectroscopy (fNIRS) and Inertial Measurement Unit (IMU) data to compare simultaneously recorded cortical activation and movement kinematics data during a reach-and-clean-up task between autistic and non-autistic children. Additionally, we examined correlations between kinematic and fNIRS data for both groups of children. 

Using parent-reported questionnaires and standardized motor assessments, approximately 50 to 88% of autistic children were found to exhibit motor difficulties [3,4,5,21,22]. These difficulties encompass atypical postural control, motor incoordination, and impairments in goal-directed movements [6,23,24]. Kinematic analyses further revealed that autistic children demonstrate inefficient movement patterns during relatively simple reaching tasks, suggesting difficulties in motor planning and execution [25,26]. For example, increased movement velocity and acceleration, as well as jerkier movement trajectories (i.e., longer movement displacements and greater movement units) have been reported in autistic children compared to non-autistic children during a solo reach-and-clean-up task, indicating impairments in both feedback and feedforward motor control mechanisms [26]. More importantly, using deep learning algorithms to reaching kinematics, it is possible to classify autistic vs. non-autistic children with 78%-92% accuracy [27], further emphasizing the sensitivity and translational potential of motor measures and their applications in identifying ASD.

Beyond solo movements, autistic children also experience difficulties moving in social contexts, such as interpersonal synchrony and imitation [10,11]. A recent meta-analysis of interpersonal synchrony reported small to large effect sizes for reduced motor synchrony in autistic children compared to those without ASD [9]. Investigating the mechanisms underlying these ASD-related social motor difficulties may provide critical insights into the links between motor impairments and the core social communication impairments in autistic children. In the current study, we expanded our previous reach-and-clean-up paradigm [26] to include an interpersonal synchrony condition and employed fNIRS to simultaneously record cortical activation along with measures of reaching kinematics.

With recent advances in neuroimaging methodologies, an increasing number of studies have been able to record cortical activation during social motor tasks [28]. A meta-analysis of Functional Magnetic Resonance Imaging (fMRI) studies reported increased neural activity in the Inferior Parietal Lobule (IPL), along with atypical activation in the occipital cortex, dorsolateral prefrontal cortex, and cingulate cortex in autistic children during action observation and imitation tasks [16]. Beyond fMRI, neuroimaging tools that impose fewer physical constraints (e.g., fNIRS) have enabled the examination of cortical activation during naturalistic social motor behaviors [29]. A recent meta-analysis of fNIRS studies focusing on social motor behaviors in autistic children revealed increased IPL activation accompanied by decreased activation in the Superior Temporal Sulcus (STS) during social motor movements compared to non-ASD peers [17]. Consistent with these findings, our research group observed increased IPL activation and reduced STS and Middle and Inferior frontal activation in autistic children during a reach-and-clean-up task [13]. Social motor impairments in ASD have been attributed to differences in mirror neuron activation in the Inferior Frontal Gyrus (IFG), STS, and IPL regions, as well as executive functioning impairments in the central executive network, including the dorsolateral prefrontal cortex, Middle Frontal Gyrus (MFG), IPL, and posterior parietal cortices; however, findings across studies remain inconsistent [17,18,30,31]. In the current study, we focused on the mirror neuron system (IFG, STS, IPL), executive functioning networks (MFG), and motor planning/control regions (pre- and post-central gyrus, PCG), which have been shown to be affected in autistic children. Additionally, we integrated detailed movement kinematics with neural measures to provide a more comprehensive understanding of how atypical neural activation patterns may contribute to social motor performance in autistic children.

Therefore, the aim of the current study is to investigate the behavioral and neural mechanisms underlying interpersonal synchrony in autistic children using simultaneous kinematic and fNIRS activation recordings. We hypothesize that autistic children will exhibit atypical kinematic profiles and altered neural activation in the IFG, STS, and IPL regions compared to non-ASD peers during both solo and joint motor tasks. Furthermore, we hypothesize that kinematic and neural measures will be significantly associated, reflecting coupling between movement performance and cortical activation. By directly linking fNIRS cortical activation with simultaneously recorded kinematic measures, this work aims to advance our understanding of neural mechanisms of atypical social motor performance and will assist in the development of objective biomarkers and intervention targets to improve social motor functioning in autistic children.

## 2. Materials and Methods

In the following paragraphs, we describe the participants, experimental procedures, equipment used for data collection, spatial registration approach, data processing, and statistical analyses.

### 2.1. Participants

Fifty-eight autistic and non-autistic children participated in the study (mean age ± standard error (SE): ASD group: 10.2 ± 0.5, 7 females, 25 males; non-ASD group: 9.9 ± 0.5, 11 females and 15 males, *p*s > 0.05; Table 1). Participants were recruited via online announcements, phone calls, and flyers distributed through ASD advocacy groups, local schools, autism services, and community centers. Prior to participation, screening interviews were conducted by phone to confirm eligibility and collect demographic information (age, sex, race, and ethnicity). Autistic children were included if they (1) were between 6 and 17 years of age, and (2) provided clinical records of confirmed ASD diagnosis, including a school record (e.g., Individualized Education Plan), and/or a medical or neuropsychological record from a psychiatrist or clinical psychologist, using the Autism Diagnostic Observation Schedule or Autism Diagnostic Interview-Revised. For the ASD group, we did not exclude children with comorbid conditions (e.g., attention-deficit/hyperactivity disorder). However, children were excluded if they (1) were unable to follow one-step instructions (e.g., “can you do this”) or (2) had significant sensory and behavioral issues preventing them from task participation or wearing the fNIRS cap. Non-autistic children (also termed non-ASD group throughout) between 6 and 17 years old without any neurological or developmental diagnoses/delays, preterm birth, significant birth history, or family history of ASD were included.

In addition to the screening interview, parents completed the Social Communication Questionnaire (SCQ; mean ± SE: 16.1 ± 1.0) [32] to screen for ASD-related social communication symptoms, as well as the Hollingshead scale for Socioeconomic Status (SES-Child; mean ± SE: ASD: 64.9 ± 3.3, non-ASD: 63.1 ± 4.1; *p* > 0.05) [33] and Coren’s handedness survey (mean ± SE: ASD: 30.9 ± 1.1, non-ASD: 33.5 ± 1.3; *p* > 0.05) [34]. Parents were also asked to complete questionnaires assessing adaptive and social functioning, including the Vineland Adaptive Behavioral Scales, 2nd edition (VABS) [35]; Social Responsiveness Scale (SRS) [36]; and Interpersonal Communication Scales (ICS) [37]. Autistic children demonstrated significantly lower VABS, SRS, and ICS scores compared to non-ASD peers (*p*s < 0.05; Table 1), indicating poorer adaptive functioning, social responsiveness, and interpersonal communication skills. Written informed consent was obtained from all parents, and assent was obtained from all participating children prior to study participation. All experimental procedures were conducted in accordance with protocols approved by the University of Delaware Institutional Review Board.

### 2.2. Experimental Procedures

**Figure 1:**
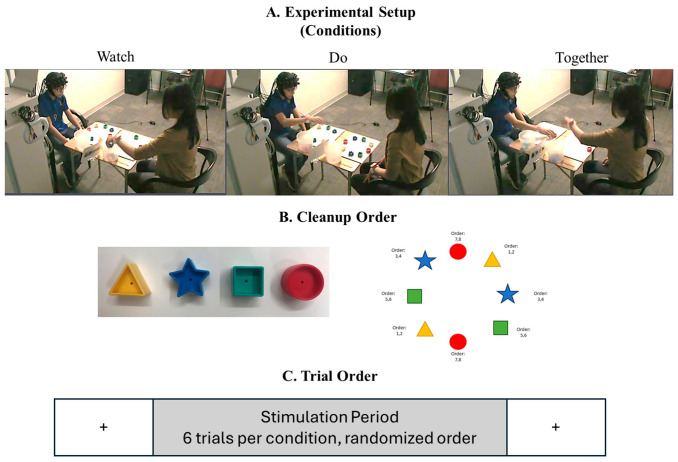
Experimental setup (**A**), clean-up order (**B**), and (**C**) trial order. Note that clean-up order varied across trials based on the picture card shown, and the child had to pick two blocks of the same color consecutively. Trial order varied in each of the 6 blocks (WDT, TWD, etc.). Written permission for publication of participant pictures was received.

During the experiment, the child sat across a table from an adult tester, with two sets of blocks arranged in a circular configuration on the table between them (Figure 1A). An fNIRS cap embedded with a 3 × 11 probe set was placed on the child’s head, covering the frontal, temporal, and parietal regions. The children completed tasks under three conditions (Figure 1A). In the Watch or “W” condition, the child observed the tester pick up the blocks one by one and place them into a container. To ensure sustained attention, the child was asked questions related to the task at the end of each trial (e.g., “Which block did I pick up first?” “Which color block did I pick up last?”). In the Do or “D” condition, the child was shown a picture card depicting block-pickup patterns and was instructed to pick up the blocks accordingly (Figure 1B). In the Together or “T” condition, the child cleaned up the blocks while following the lead of the adult tester, synchronizing both block position and the timing of block pickup and drop into the container.

Ten seconds before and after each clean-up trial is considered the baseline period, during which the child was instructed to observe a crosshair and remain still to allow the hemodynamic response to return to baseline levels (Figure 1C). We followed a randomized block design, wherein each child completed a total of 18 trials (6 trials per condition), which were randomized in blocks of 3 trials (WDT, TWD, etc.), over the course of the single fNIRS session. All participants were tested in the same environment. We ensured that the setup was consistent across participants, including standardized lighting and closing the door to minimize external noise.

### 2.3. Data Collection

**Figure 2:**
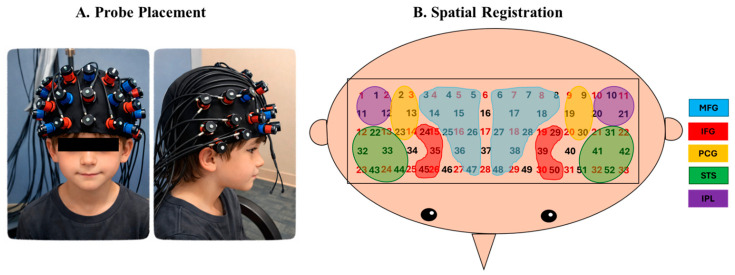
Probe placement (**A**) and spatial registration output (**B**).

Hemodynamic changes during the block clean-up task were recorded using a Hitachi ETG-4000 fNIRS system (Hitachi Medical Systems, Inc., Schaumburg, IL, USA; sampling rate: 10 Hz). A cap embedded with a 3 × 11 fNIRS probe set was placed on the child’s head, covering bilateral frontal, temporal, and parietal regions (Figure 2A). To ensure consistent probe placement across participants, the middle column of the probe set was aligned with the child’s nasion and the bottom row with the child’s brow line during cap placement (Figure 2A). The probe set consisted of 17 infrared emitters and 16 receivers, with each adjacent emitter–receiver pair separated by 3 cm. The emitters delivered infrared light at two wavelengths (695 and 830 nm). Infrared light passed through the scalp and skull in a banana-shaped trajectory, reaching cortical tissue approximately beneath the midpoint between each emitter–receiver pair. Each emitter–receiver midpoint was defined as a measurement channel, resulting in a total of 52 channels across the probe set. Changes in the absorption and attenuation of infrared light were used to calculate concentrations of oxygenated (HbO_2_) and deoxygenated hemoglobin (HHb) for each channel using the modified Beer–Lambert law [29]. Increased cortical activation within a given region of interest was typically associated with an increase in HbO_2_ and a concurrent decrease in HHb concentration [29]. In addition to the fNIRS probe set, an inertial measurement unit (IMU; Xsens, Inc., El Segundo, CA, USA) was placed on the child’s right wrist to record x-, y-, and z-axis displacement of hand movements. E-Prime presentation software (version 2.0) was used to trigger both the Hitachi fNIRS and IMU systems. The entire session was videotaped using a camcorder (Sony Corporation, Tokyo, Japan) that was synchronized with the Hitachi fNIRS system.

### 2.4. Spatial Registration Approach

After fNIRS data collection, 3D registration of probe placement was performed using the ETG-4000 3D positioning unit (Hitachi Medical Corporation, Schaumburg, IL, USA) with the Polhemus motion tracking system (Polhemus Inc., Lake Mary, FL, USA). Children were asked to remain seated and still while the Polhemus system recorded the 3D coordinates of cranial landmarks (including the nasion, inion, left and right preauricular points, and the Cz position defined by the international 10–20 system), as well as the locations of each emitter and receiver. All coordinates were recorded in a reference coordinate system and subsequently transformed into Montreal Neurological Institute (MNI) space using an anchor-based spatial registration method developed by our collaborator [38]. By integrating structural information from an anatomical database [39], channel positions were estimated within a standardized 3D brain atlas [38]. Channel locations were then averaged across participants and labeled using the LONI Probabilistic Brain Atlas (LPBA) [40].

A channel was included in a Region of Interest (ROI) if more than 60% of its probabilistic overlap fell within that ROI and was excluded if its homologous channel was assigned to a different ROI. Five ROIs were defined (Figure 2B): (i) Middle Frontal Gyrus (MFG; left: channels 6, 7, 8, 17, 18, 27, 28, 38, 48; right: 3, 4, 5, 14, 15, 25, 26, 36, 47); (ii) Inferior Frontal Gyrus (IFG; left: 29, 39, 50; right: 24, 35, 45); (iii) Precentral and Postcentral Gyri (PCG; left: 9, 19, 30; right: 2, 13, 23); (iv) Superior Temporal Sulcus (STS; left: 31, 41, 42, 51, 52; right: 22, 32, 33, 43, 44); and (v) Inferior Parietal Lobule (IPL; left: 10, 20, 21; right: 1, 11, 12). Channels 16 and 37 were excluded because they were located at the midline, and channels 34, 40, 46, and 49 were excluded because their homologous channels were assigned to different ROIs. Detailed channel assignments are provided in Supplementary Table S1.

### 2.5. Data Processing

Kinematic data recorded by the IMU sensor captured wrist displacement in a three-dimensional coordinate system. Raw displacement data from each participant were first transformed into a common reference coordinate system. Movement onset was identified when both the relative displacement (with respect to the starting position) and acceleration reached 20% of their respective maximum values. The following kinematic parameters were derived from the IMU data collected during the block clean-up task during the Do and Together conditions: (A) Reaction time was defined as the interval between the task initiation signal and movement onset, (B) movement time was defined as the interval between movement onset and task completion, (C) displacement was defined as the total distance traveled by the hand to complete the task, (D) average velocity was defined as the mean velocity across the entire task period, (E) average acceleration was defined as the mean acceleration throughout the task period, and (F) movement units were defined as the number of discrete movement segments identified by zero crossings in acceleration for the entire task period. Raw acceleration data were used for zero-crossing analysis due to the relatively large movement amplitudes, which minimize the impact of high-frequency noise on the measures of interest. To integrate zero-crossing events across the three spatial axes, root mean squared (RMS) values were calculated for each movement unit measure.

For cortical activation analyses, we developed custom MATLAB (Mathworks Inc., Natick, MA, USA; version 23.2) scripts that incorporated the open-source software packages Homer-2 [41] and Hitachi PoTATo (version 3.8) [42] to process the fNIRS signal. Raw signals were band-pass filtered between 0.01 and 0.05 Hz to remove physiological noise (e.g., respiration and cardiac signals) as well as environmental noise (e.g., ambient light). Motion artifacts were corrected using a wavelet-based method [41,43]. A General Linear Model (GLM) incorporating Gaussian basis functions and a third-order polynomial drift regression was then applied to estimate the hemodynamic response function [41].

**Figure 3:**
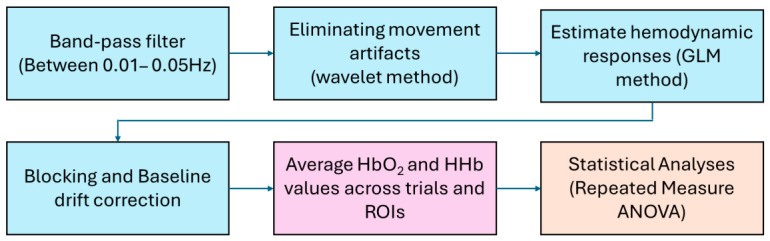
fNIRS data processing flowchart.

To correct for baseline drift over the course of the experiment, linear trends between the pre- and post-stimulation fixation periods were subtracted from the corresponding clean-up periods [42]. Finally, oxygenated (HbO_2_) and deoxygenated (HHb) hemoglobin concentrations were averaged for each task block. The fNIRS data processing flowchart is presented in Figure 3. In the Section 3, we focus on HbO_2_ responses, as HbO_2_ typically exhibits a larger dynamic range and higher signal-to-noise ratio than HHb [43].

### 2.6. Statistical Analyses

For reaching kinematics, two-way ANOVAs were conducted with condition (Watch, Do, Together) as the within-subjects factor and group (non-ASD, ASD) as the between-subjects factor. For the fNIRS data, channels within the same ROI were averaged to reduce the number of comparisons. Linear mixed-effects models were used to examine the effects of group, condition, hemisphere regions and their interactions on HbO_2_ concentration, with participant included as a random intercept to account for repeated measures within individuals. When the assumption of sphericity was violated, as indicated by Mauchly’s test, Greenhouse–Geisser corrections were applied.

In addition, Pearson correlation analyses were conducted to examine the relationships among cortical activation, VABS scores, and kinematic measures. To correct for multiple comparisons from all analyses, the false discovery rate (FDR) procedure was applied to control for Type I error [44]. Specifically, unadjusted *p*-values from all post hoc tests were rank-ordered from smallest to largest, and results were considered statistically significant if the unadjusted *p*-value was less than the corresponding FDR-adjusted threshold, calculated as 0.05 × (i/n), where i is the rank order of the *p*-value and n equals total number of comparisons. FDR corrections were performed separately for each set of correlations (i.e., correlations between cortical activation and VABS scores, and correlations between reaching kinematics and cortical activation). All statistical analyses were conducted using IBM SPSS Statistics (version 29) (IBM Corp., Armonk, NY, USA).

## 3. Results

In the following paragraphs, we present the reaching kinematics and cortical activation associated with the reach-to-clean-up task. Additionally, we report correlations between cortical activation and children’s VABS scores, as well as between cortical activation and reaching kinematics.

### 3.1. Reaching Kinematics

**Figure 4:**
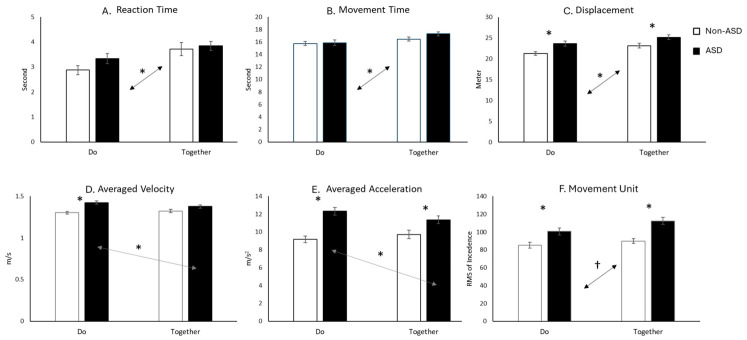
Reaching kinematics, including reaction time (**A**), movement time (**B**), displacement (**C**), averaged velocity (**D**), averaged acceleration (**E**), and number of movement units (**F**). † indicates *p* < 0.05 but did not survive FDR corrections. * indicates *p* < 0.05 and survived FDR corrections. Bidirectional arrows indicate the direction of comparison.

The linear mixed-effects model revealed a significant main effect of condition on reaction time (F (1, 435) = 9.68, *p* < 0.05, Figure 4A), movement time (F (1, 435) = 6.18, *p* < 0.05, Figure 4B), and displacement (F (1,435) = 6.93, *p* < 0.05, Figure 4C). Children in both groups exhibited significantly longer reaction time, movement time, and displacement during the Together condition compared to the Do condition (*p*s < 0.05). Additionally, significant main effects of group were observed for displacement (F (1, 435) = 10.08, *p* < 0.01) and movement units (F (1, 435) = 22.49. *p* < 0.001), with autistic children showing longer displacement and a higher number of movement units compared to their non-autistic peers (*p*s < 0.05, Figure 4C,F). Both average velocity and average acceleration showed borderline significant group x condition interactions (average velocity (F (1, 435) = 3.20, *p* = 0.075; average acceleration (F (1, 435) = 3.10, *p* = 0.081, Figure 4D,E). For average velocity, autistic children moved faster than non-autistic children in the Do condition (*p* < 0.05), whereas no group differences were observed in the Together condition (*p* > 0.05). Within the ASD group, average velocity was lower in the Together condition compared to the Do condition (*p* < 0.05), while no significant condition-related differences were found in the non-ASD group (*p* > 0.05). For averaged acceleration, autistic children showed significantly greater changes in speed than the non-ASD group in both the Do and Together conditions (*p*s < 0.05). However, only autistic children showed a reduction in average acceleration (i.e., fewer speed changes) in the Together condition compared to the Do condition (*p* < 0.05).

### 3.2. Cortical Activation

The linear mixed-effects model revealed a significant main effect of condition (*F* (2, 12,384) = 37.80, *p* < 0.001), hemisphere (*F* (1, 12384) = 10.09, *p* = 0.001), and region (*F* (5, 12,384) = 164.78, *p* < 0.001). Significant two-way interactions were observed for group × hemisphere (*F* (1, 12,384) = 20.57, *p* < 0.001), group × region (*F* (5, 12,384) = 5.40, *p* < 0.001), condition × hemisphere (*F* (2, 12,384) = 3.06, *p* < 0.05), and hemisphere × region (*F* (5, 12,384) = 16.68, *p* < 0.01). In addition, a significant three-way interaction among group, hemisphere, and region was found (*F* (5, 12,384) = 6.30, *p* < 0.001). Based on these findings, we further examined the group × hemisphere × region interaction, as well as the main effects of condition.

#### 3.2.1. Group-Related Differences in Cortical Activation

**Figure 5:**
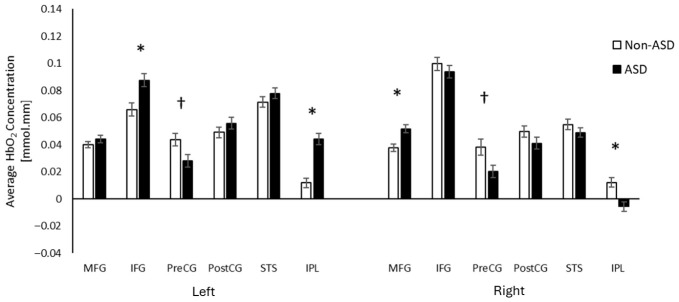
Group differences in cortical activation (averaged HbO_2_). * Indicates *p* < 0.05 and survived FDR corrections; † indicates *p* < 0.05 but did not survive FDR corrections.

Post hoc analyses of the three-way interaction showed hyperactivation in the left IFG, left IPL, and right MFG in the ASD group compared to the non-ASD group (*p*s < 0.001, survived FDR corrections; Figure 5). Additionally, the ASD group showed a trend toward hypoactivation in the bilateral preCG (*p*s < 0.05, did not survive FDR correction) as well as the right IPL (*p* < 0.001, survived FDR corrections; Figure 5). Detailed t-statistics can be found in Supplementary Table S2.

#### 3.2.2. Hemispheric Differences in Cortical Activation

**Figure 6:**
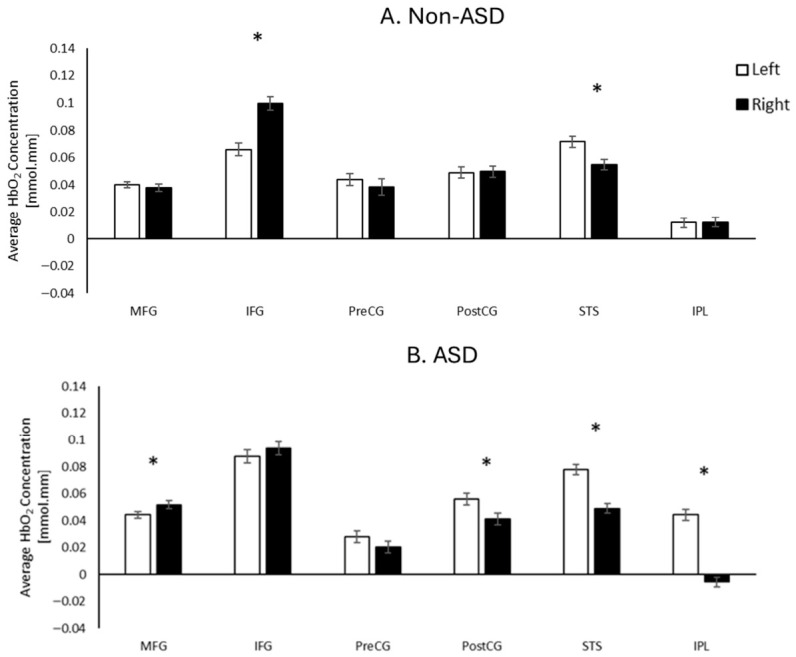
Hemispheric differences in cortical activation (averaged HbO_2_) in the non-ASD (**A**) and ASD (**B**) groups. * Indicates *p* < 0.05 and survived FDR corrections.

Post hoc analyses of the three-way interaction revealed distinct hemispheric differences in activation patterns in both groups (Figure 6). In the non-ASD group, right IFG activation was greater than left IFG (*p*s < 0.01, survived FDR correction). In contrast, left STS activation was greater than right STS (*p*s < 0.001, survived FDR correction). In autistic children, right MFG activation was greater than left MFG (*p* = 0.004, survived FDR correction). Additionally, autistic children demonstrated greater activation in the left hemisphere compared to the right hemisphere in the post-CG, STS, and IPL regions (*p*s < 0.001, survived FDR corrections). Detailed t-statistics can be found in Supplementary Table S2.

#### 3.2.3. Condition-Related Differences in Cortical Activation

Post hoc analyses of the main effect of condition revealed a sequential increase in cortical activation from Watch to Do to Together. Specifically, activation was significantly greater during Do compared to Watch (*p* < 0.001, survived FDR correction), Together compared to Watch (*p* < 0.001, survived FDR correction), and Together compared to Do (*p* < 0.001, survived FDR correction). Detailed t-statistics are provided in Supplementary Table S3.

### 3.3. Correlating Cortical Activation and VABS Performance

Correlation analyses between cortical activation and VABS scores revealed negative correlations in the non-ASD group but positive correlations in the autistic children (Table 2). Specifically, in the non-ASD group, higher VABS scores were associated with lower activation in the left IFG and right pre-CG during the Watch condition (r = −0.314 and −0.385, *p*s < 0.01) and lower activation in the left MFG and left IPL during the Together condition (r = −0.333 and −0.427, *p*s < 0.01). In contrast, in autistic children, higher VABS scores were associated with greater activation in the left STS and left IPL during the Watch condition (r = 0.297 and 0.341, *p*s < 0.01), greater left IPL activation during the Do condition (r = 0.384, *p* < 0.01), and higher activation in the left pre-CG and left IPL ROIs during the Together condition (r = 0.296 and 0.238, *p*s < 0.01).

### 3.4. Correlating Reaching Kinematics with Cortical Activation

Correlations revealed distinct neuro-motor associations across conditions in the non-ASD group and autistic children. The non-ASD group had more associations during the Do versus the Together condition (Table 3). During the Do condition, greater left post-CG activation was significantly associated with longer reaction times (r = 0.367, *p* < 0.01), while lower left IPL activation was associated with longer arm movement times (r = −0.319, *p* < 0.01). Additionally, greater right IFG activation was associated with a higher movement unit count (r = 0.330, *p* < 0.01), whereas greater right pre-CG activation was associated with both longer movement times (r = 0.342, *p* < 0.01) and higher movement unit count (r = 0.481, *p* < 0.01). No significant correlations with *p*-values lower than 0.01 were observed during the Together conditions.

In autistic children, during the Do condition, greater left STS activation was significantly associated with longer movement times (r = 0.244, *p* < 0.01), while greater right pre-CG activation correlated with longer displacements (r = 0.219, *p* < 0.01). Furthermore, increased right IPL activation was linked to greater displacement (r = 0.231, *p* < 0.01), average velocity (r = 0.242, *p* < 0.01), and average acceleration (r = 0.275, *p* < 0.01). During the Together condition, greater left MFG activation was associated with lower average acceleration (r = −0.221, *p* < 0.01), while greater right MFG activation correlated with longer movement times (r = 0.227, *p* < 0.01). Additionally greater left post-CG activation was significantly associated with higher movement unit count (r = 0.243, *p* < 0.01). Greater right IFG activation was linked to lower average velocity (r = −0.238, *p* < 0.01) and averaged acceleration (r = −0.267, *p* < 0.01). Lastly, greater right pre-CG activation was correlated with both longer displacement (r = 0.296, *p* < 0.01) and higher movement unit count (r = 0.260, *p* < 0.01).

## 4. Discussion

A high proportion of autistic children exhibit motor challenge that substantially limits their participation in daily activities [3,6,8]. While previous studies have separately reported behavioral and neural characteristics of autistic children, the links between them remain largely unexplored. In the current study, we simultaneously recorded fNIRS-based neural activity and IMU-based arm movement kinematics during a reach-and-clean-up task to better elucidate the behavioral and neural mechanisms underlying ASD-related motor difficulties.

Our findings revealed atypical reaching kinematics in autistic children, characterized by longer movement displacements, higher average velocity and acceleration, and a greater number of movement units, compared to the non-ASD group. Compared to the non-ASD group, autistic children also exhibited atypical cortical activation, including hypoactivation in the STS and pre-CG, along with hyperactivation in the MFG, IFG, and IPL. Furthermore, patterns of hemispheric lateralization differed between autistic and non-autistic children, suggesting engagement of different neural networks during goal-directed motor behavior. These cortical activation patterns were significantly associated with both adaptive functioning and kinematic characteristics, pointing to a unique compensatory neural strategy in autistic children. Together, these findings extend prior neuroimaging and kinematic findings by directly linking neural activation to motor behaviors of autistic children, thereby providing deeper insight into the mechanisms underlying their motor difficulties. This integrative approach has the potential to inform the development of better screening tools and targeted intervention strategies for autistic children.

### 4.1. Kinematic Evidence for Reduced Feedforward Control and Increased Reliance on Feedback in Autistic Children

During the reach-and-clean-up task, both groups exhibited longer reaction times, longer movement times, greater displacement, and a greater number of movement units in the Together condition compared with the Do condition, indicating that both groups adapted their movement patterns to comply with the instructions to move together. However, compared to the non-ASD group, autistic children exhibited greater movement overshooting (indicated by longer movement displacement), higher movement velocity and acceleration, and a greater number of movement units during the Do and/or Together conditions. These findings are consistent with previous kinematic studies reporting atypical motor control in autistic children [23,45].

Specifically, Cook et al. (2013) found increased movement acceleration and velocity, along with a greater number of movement units (i.e., jerkier movement) during horizontal sinusoidal movements [45]. Similarly, Chen et al. (2019) reported greater shoulder and elbow movement amplitude and velocity, as well as an increased number of movement units during a ball-catching task [23]. Autistic children may have difficulty integrating successive movement components into an action sequence; instead, they may execute each movement segment as an independent movement [46,47]. This segmentation may result in less precise predictive control over movement termination, including when to slow down or change direction, leading to autistic children relying more heavily on feedback-based adjustments [46,47,48]. The repetitive and rhythmic nature of the current reach-and-clean-up task requires children to anticipate subsequent reaching movements and adjust movement speed and direction accordingly, thereby placing increased demands on the coordination of movement sequences. The combination of increased movement overshooting, elevated velocity and acceleration, and a greater number of movement units suggests reduced feedforward control and a greater reliance on online corrective processes during goal-directed movements [46,47,48].

### 4.2. Altered Motor Cortical and Frontoparietal Activation Supporting Atypical Feedforward–Feedback Control in ASD

In terms of cortical activation, autistic children demonstrated hypoactivation in bilateral pre-CG and right IPL, along with hyperactivation in the right MFG, left IFG, and left IPL ROIs across conditions. Given the spatial resolution of fNIRS, the pre-CG ROI likely includes the primary motor cortex (M1), premotor, and supplementary motor areas that support the planning and execution of both internally and externally guided movement sequences [49]. In contrast, the MFG and IFG are associated with executive functions such as working memory and inhibition [50,51], while the IPL contributes to encoding movement kinematics, particularly during internally guided actions [52,53]. During the Do condition, the block pick-up and drop-off actions occurred sequentially, requiring internally guided movement sequences, while the Together condition further required the participant to monitor the tester’s actions and synchronize their movements with the tester; making it more externally guided.

The finding of reduced pre-CG activation in autistic children may reflect less efficient feedforward motor control. Instead of executing a smooth movement sequence, autistic children may have segmented each action component, increasing reliance on frontoparietal regions to support online adjustments and control. Consistent with this interpretation, activation in the pre-CG, MFG, IFG, and IPL during the Do and/or Together conditions was significantly associated with kinematic measures, directly linking cortical activation to motor performance. This pattern suggests reduced feedforward control and greater reliance on feedback-control mechanisms and cognitively mediated compensation in autistic children. Moreover, greater IPL activation was associated with higher adaptive functioning in autistic children. This finding is consistent with recent meta-analytic evidence and suggests that increased IPL recruitment may also reflect a compensatory neural mechanism [17].

Alternatively, increased activation in the frontoparietal regions may reflect greater cognitive effort, inefficient motor planning, and/or altered sensorimotor integration in autistic children. Previous studies have reported increased frontal and parietal activation measured by fNIRS during tasks with higher cognitive demands (e.g., 2-back vs. 1-back), suggesting that frontoparietal regions are sensitive to cognitive load [54]. In addition, prefrontal hyperactivation has been linked to less efficient motor planning and has been reported in older adults [55,56]. The parietal cortex, which plays a key role in sensorimotor processing [57], has also shown atypical activation patterns in children with developmental coordination disorder [58]. Taken together, heightened frontoparietal activation may reflect a compensatory neural response, potentially indicating increased task-related demands along with less efficient motor planning and altered sensorimotor processing in autistic children.

### 4.3. Atypical Lateralization in Cortical Activation in Autistic Children

The current study found similar condition-related differences in autistic and non-autistic children, characterized by a sequential increase in cortical activation from Watch to Do to Together. These findings are consistent with prior literature, suggesting greater cortical involvement during action execution compared to observation, and greater cortical involvement during interpersonal synchrony compared to moving alone [13]. Moreover, this finding supports the idea that autistic children have fundamental differences in controlling movements (solo and social) and the underlying mechanisms versus specific social motor differences, compared to those without ASD. Regarding hemispheric specialization, both groups exhibited right lateralization in frontal regions (MFG and/or IFG) and left lateralization in the STS ROI. However, autistic children additionally demonstrated left lateralization in the post-CG and IPL. The post-CG encompasses the primary somatosensory cortex and plays a critical role in sensorimotor control, while the IPL is involved in planning movement kinematics [52,53,59,60]. Given that the task was performed using the right hand, left-hemispheric post-CG and IPL engagement may reflect increased contralateral recruitment to support kinematic planning and sensorimotor control. These findings suggest atypical neural network allocation and hemispheric activation in autistic children, characterized by less reliance on motor cortices and greater engagement of frontoparietal control systems (i.e., with greater cognitive awareness) during goal-directed movements and interpersonal synchrony compared to non-autistic controls.

### 4.4. Limitations and Future Directions

Although we explored differences across observation, execution, and joint-action conditions, it did not permit a clear dissociation of feedforward versus feedback control processes, nor did it independently isolate internally and externally guided movement mechanisms. Future studies should employ paradigms that directly manipulate sensory perturbations or cue predictability to more precisely characterize the underlying control mechanisms. Secondly, while fNIRS offers advantages for pediatric and ecologically valid research contexts, its limited temporal resolution constrains the ability to resolve rapid neural dynamics and their coupling with kinematic changes. Multimodal approaches, such as integrating fNIRS with EEG alongside motion capture, would enhance temporal precision and facilitate a more comprehensive understanding of brain–behavior relationships underlying social motor difficulties in autistic children. Lastly, trial-level correlations were conducted to examine the relationship between cortical activation and movement kinematics within the same trial. However, this approach may inflate the effect sizes of associations; therefore, the correlation findings should be interpreted with caution.

### 4.5. Clinical Implications

The current study suggests potential neural and kinematic biomarkers in autistic children that may contribute to improved screening, particularly following validation in larger and younger samples. For example, with the application of machine learning approaches, neural and kinematic features may serve as a supplement to existing diagnostic methods to facilitate the identification of autistic children [26,27]. In clinical settings, therapists often use social motor-based interventions, such as music and movement, dance, and hippotherapy, to improve social motor difficulties in autistic children [61,62,63]. Neural and kinematic measures may serve as objective outcome indicators to track intervention-related changes over time [63,64,65].

## 5. Conclusions

Using simultaneous recordings of neural and kinematic measures, the present study identified distinct neural activation and behavioral patterns associated with social motor performance in autistic children. Autistic children exhibited increased movement segmentation and a greater reliance on online feedback control during task execution. In terms of cortical activation, they showed reduced engagement of motor-related cortical regions and increased activation in frontoparietal control networks, suggesting greater cognitive involvement during movement planning and execution. Together, these interconnected neural and behavioral findings may inform the development of more sensitive ASD screening tools and support the design of targeted motor interventions for autistic children with fNIRS activation patterns as neural correlates that are amenable to change post-intervention.

## Figures and Tables

**Table 1:** Participant demographic information.

Characteristics	ASD Group (n = 32) Mean ± SE	Non-ASD Group (n = 26) Mean ± SE
Age	10.2 ± 0.5	9.9 ± 0.5
Sex	7 F, 25 M	11 F, 15 M
Race	20 C, 3 A, 6 AA, 3 MR	16 C, 3 A, 1 AA, 6 MR
Ethnicity	5 H, 27 NH	2 H, 24 NH
SES-Child	64.9 ± 3.3	63.1 ± 4.1
Coren’s handedness score	28 RH, 4 LH 30.9 ± 1.1	24 RH, 2 LH 33.5 ± 1.3
SCQ	16.1 ± 1.0	-
VABS-II (SS)		
Communication	82.1 ± 2.6 *	106.2 ± 2.8
Daily living	80.2 ± 2.4 *	101.5 ± 3.2
Socialization	72.7 ± 2.6 *	103.2 ± 3.9
Total	76.3 ± 2.2 *	103.7 ± 3.0
SRS (T-score)	73.6 ± 1.6 *	55.5 ± 1.7
ICS	4.8 ± 0.1 *	5.4 ± 0.1

ASD = Autism Spectrum Disorder; non-ASD = non-autistic children; SE = standard error; F = female; M = male; C = Caucasian, A = Asian, AA = African American; MR = mixed race; H = Hispanic; NH = Non-Hispanic; SES-Child = Hollingshead Four-Factor Index of Socioeconomic Status; RH = right-handed; LH = left-handed; SCQ = Social Communication Questionnaire; VABS-II = Vineland Adaptive Behavior Scale, 2nd Edition; SS = standard score; SRS = Social Responsiveness Scale; ICS = Interpersonal Communication Scale; * indicates significant differences between ASD and non-ASD groups.

**Table 2:** Correlations between cortical activation and VABS score.

	Non-ASD			ASD		
	Watch	Do	Together	Watch	Do	Together
Left MFG	−0.220	−0.203	−0.333 **	−0.161	−0.183 *	−0.141
Left IFG	−0.314 **	−0.199	−0.204	−0.050	−0.036	0.059
Left PreCG	−0.083	0.094	−0.191	0.090	0.025	0.296 **
Left PostCG	0.208	0.171	−0.241 *	0.123	0.009	−0.051
Left STS	−0.001	0.131	−0.077	0.297 **	0.205 *	0.159
Left IPL	0.078	0.059	−0.427 **	0.341 **	0.384 **	0.238 **
Right MFG	−0.042	0.108	0.032	0.077	0.106	0.154
Right IFG	−0.179	−0.234	−0.288	0.045	−0.080	−0.184 *
Right PreCG	−0.385 **	−0.259 *	−0.170	0.084	0.070	0.406
Right PostCG	−0.290 *	0.033	−0.190	0.126	0.124	0.069
Right STS	0.048	0.024	0.035	0.157	0.139	0.072
Right IPL	−0.045	0.115	−0.067	0.202 *	0.032	0.021

* *p* < 0.05, ** *p* < 0.01. False discovery rate (FDR) correction was applied across 72 comparisons in this table. Shaded cells indicate results that survived FDR correction.

**Table 3:** Correlations between reaching kinematics and cortical activation.

	Reaction Time	Movement Time	Displacement	Averaged Velocity	Averaged Acceleration	Movement Unit
Non-ASD group						
Do condition						
Left MFG	0.0091	0.123	−0.033	−0.267 *	−0.271 *	−0.093
Left IFG	0.181	−0.012	0.049	−0.008	−0.133	−0.270 *
Left PreCG	0.097	−0.039	−0.125	−0.151	−0.110	−0.026
Left PostCG	0.367 **	0.061	0.067	−0.036	−0.099	−0.118
Left STS	0.131	−0.151	−0.031	0.103	0.105	−0.163
Left IPL	0.056	−0.319 **	−0.109	0.222	0.283 *	−0.007
Right MFG	0.080	0.198	0.143	−0.101	−0.077	0.210
Right IFG	0.051	0.218	0.146	−0.104	−0.013	0.330 **
Right PreCG	−0.221	0.342 **	0.198	−0.158	−0.059	0.481 **
Right PostCG	0.161	−0.273 *	−0.162	0.080	0.069	−0.182
Right STS	0.190	−0.200	−0.084	0.087	0.028	−0.240 *
Right IPL	−0.007	−0.233	−0.131	0.095	0.085	−0.153
Together condition						
Left MFG	−0.036	0.022	−0.120	−0.208	−0.192	−0.011
Left IFG	−0.177	0.128	−0.015	−0.133	−0.211	−0.180
Left PreCG	−0.040	0.115	0.093	0.014	−0.021	0.131
Left PostCG	−0.139	0.169	0.004	−0.127	−0.168	−0.006
Left STS	0.270 *	0.179	0.077	−0.020	−0.009	0.067
Left IPL	0.154	0.001	0.246 *	0.285 *	0.233 *	0.195
Right MFG	0.030	0.003	−0.009	−0.048	−0.007	0.136
Right IFG	0.165	−0.085	0.098	0.170	0.215	0.238 *
Right PreCG	0.168	0.043	−0.049	−0.137	−0.124	0.163
Right PostCG	−0.098	−0.132	0.024	0.190	0.143	−0.093
Right STS	0.048	−0.128	−0.035	0.047	−0.028	−0.212
Right IPL	−0.096	−0.219	−0.159	0.001	0.042	−0.128
Autistic children						
Do condition						
Left MFG	0.208 *	−0.113	−0.206 *	−0.213 *	−0.212 *	−0.060
Left IFG	0.036	0.128	0.094	−0.079	−0.151	0.011
Left PreCG	0.018	−0.035	−0.060	−0.077	−0.097	−0.027
Left PostCG	0.107	−0.062	−0.037	0.020	0.006	−0.049
Left STS	0.013	0.244 **	0.201 *	−0.039	−0.074	0.135
Left IPL	0.013	−0.020	0.082	0.174 *	0.173 *	−0.019
Right MFG	0.016	0.214 *	0.132	−0.102	−0.114	0.094
Right IFG	0.086	0.004	−0.101	−0.106	−0.171 *	−0.093
Right PreCG	−0.059	0.169 *	0.219 **	0.136	0.121	0.133
Right PostCG	0.027	0.099	0.159	0.123	0.122	0.154
Right STS	0.030	0.108	0.083	−0.021	−0.038	0.072
Right IPL	−0.154	0.128	0.231 **	0.242 **	0.275 **	0.201 *
Together condition						
Left MFG	−0.065	0.105	−0.075	−0.194 *	−0.221 **	0.114
Left IFG	0.095	0.082	0.063	−0.020	−0.108	−0.059
Left PreCG	−0.015	−0.049	−0.011	0.050	0.024	0.026
Left PostCG	−0.130	0.167 *	0.058	−0.084	−0.092	0.243 **
Left STS	−0.041	0.058	0.075	0.061	0.026	0.054
Left IPL	−0.087	0.034	0.075	0.086	0.100	0.121
Right MFG	0.097	0.227 **	0.175 *	−0.014	−0.068	0.076
Right IFG	0.036	0.193 *	−0.016	−0.238 **	−0.267 **	0.113
Right PreCG	−0.045	0.210 *	0.296 **	0.166 *	0.179 *	0.260 **
Right PostCG	−0.115	0.129	−0.029	−0.134	−0.111	0.197 *
Right STS	−0.024	0.198 *	0.124	−0.034	−0.025	0.154
Right IPL	−0.137	0.100	0.074	0.003	0.039	0.120

* *p* < 0.05, ** *p* < 0.01. False discovery rate (FDR) correction was applied across 288 comparisons in this table. Shaded cells indicate results that survived FDR correction.

## Data Availability

The data presented in this study are available on request from the corresponding author. The data are not publicly available due to restrictions associated with participants’ privacy.

## Supplementary Materials

The following supporting information can be downloaded at: https://www.mdpi.com/article/10.3390/brainsci16050540/s1, Table S1: Channel assignments based on the spatial registration approach. For each channel, the spatial location in the MNI’s coordinate system and the probability of covering different brain regions are shown. The channels are symmetrically divided across the two hemispheres (left, right). The color-coded channels were considered within a specific ROI (MFG, IFG, PCG, STS, and IPL). Table S2: The post hoc analyses for the group × condition × hemisphere × region four-way interaction. Table S3. The post-hoc analyses for the main effect of Condition.

## Abbreviations

The following abbreviations are used in this manuscript:

ASD	Autism Spectrum Disorder
Non-ASD	Non-Autism Spectrum Disorder
SCQ	Social Communication Questionnaire
VABS-II	Vineland Adaptive Behavior Scale, 2nd Edition
fNIRS	Functional Near-Infrared Spectroscopy
IMU	Inertial Measurement Unit
MFG	Middle Frontal Gyrus
IFG	Inferior Frontal Gyrus
PCG	Precentral and Postcentral Gyri
STS	Superior Temporal Gyrus
IPL	Inferior Parietal Lobule
