# Supplementary material for: Neural and Kinematic Characteristics of Reaching in Autistic Children During Movement Observation, Execution, and Synchronization: An fNIRS Study
Source: Brain Sci. 2026 May 20;16(5):540. doi: 10.3390/brainsci16050540 (PMC13204447; doi:10.3390/brainsci16050540)
Supplement: Supplementary file 1 [file brainsci-16-00540-s001.zip › brainsci-4191988-supplementary.pdf]

Neural and kinematic characteristics of reaching in autistic children during movement observation, execution, and synchronization: An fNIRS study

Supplementary Table S1. Channel assignments based on the spatial registration approach (Tsuzuki et al., 2012). For each channel, the spatial location in MNI's coordinate system and the probability of covering different brain regions are shown. The channels are symmetrically divided across the two hemispheres (left, right). The color-coding depicts the specific ROI a given channel covered (MFG, IFG, PCG, STS, and IPL).

Side	CH	MNI's coordinate			MFG		IFG	PCG		STS		IPL		Assigned region
		X	Y	Z	Middle frontal gyrus	Superior frontal	Inferior frontal gyrus/OFG	Precentral gyrus	Postcentral gyrus/SMG	Superior temporal gyrus	Middle temporal gyrus/ITG	Supramarginal gyrus	Angular gyrus	
Left	6	-9.3	53.3	46.7	2.8	97.2								MFG
	7	-33.3	39.3	45.0	93.9	6.1								MFG
	8	-50.0	18.7	44.7	94.6			5.4						MFG
	9	-61.0	-8.3	42.7				30.5	69.5					PCG
	10	-65.0	-34.3	44.3								100		IPL
	17	-24.3	57.7	33.3	97.7	2.3								MFG
	18	-47.0	37.7	31.7	100.0									MFG
	19	-60.0	10.7	30.7	10.1		12.6	77.3						PCG
	20	-67.3	-18.3	31.7								67.1	32.9	IPL
	21	-66.0	-45.3	34.3								79.7	20.3	IPL
	27	-11.3	69.3	21.7	24.8	75.2								MFG
	28	-39.7	56.3	20.3	100.0									MFG
	29	-56.0	30.0	18.0	10.8		89.2							IFG
	30	-66.0	-1.3	18.7				44.2	55.8					PCG
	31	-69.0	-31.3	20.7					1.3	62.8		35.9		STS
	38	-26.3	68.7	7.3	100.0									MFG
	39	-49.7	47.7	5.3	8.2		91.8							IFG
	40	-59.7	18.3	5.7			86.8	12.7		0.5				Excluded
	41	-68.0	-13.7	4.3				15.4		68.6	16.0			STS
	42	-70.0	-43.3	6.7			11.1			40.6	59.4			STS
	48	-10.0	72.7	-6.0	9.1	90.1								MFG
	49	-38.7	62.7	-7.0	65.6		38.5							Excluded
	50	-54.0	38.3	-6.7			100.0							IFG
	51	-63.0	2.3	-10.7						64.6	35.4			STS
	52	-71.0	-26.3	-9.3							100.0			STS
	16	6.3	64.0	32.7	23.4	76.6								Excluded
	37	7.3	73.0	8.0	54.5	58.6								Excluded
Right	1	66.7	-36.7	44.3								85.5	14.5	IPL
	2	64.0	-10.7	43.3				2.7	97.3					PCG
	3	53.0	16.7	43.3	68.8		0.6	30.7						MFG

	4	38.3	39.3	44.0	100.0									MFG
	5	17.0	52.7	45.0	43.9	56.1								MFG
	11	66.0	-48.3	33.7						6.1		72.3	21.6	IPL
	12	70.0	-21.7	32.3					8.9			91.1		IPL
	13	64.0	7.7	31.7			0.3	82.4	17.3					PCG
	14	51.7	35.7	30.3	67.8		32.2							MFG
	15	30.7	56.7	31.7	100.0									MFG
	22	71.0	-34.3	20.3						62.5		24.9	12.7	STS
	23	69.0	-3.7	19.3				6.2	91.2	2.4	0.3			PCG
	24	60.0	27.3	18.3			80.3	19.7						IFG
	25	45.3	53.3	19.3	60.1		39.9							MFG
	26	21.3	69.0	21.3	99.4		0.6							MFG
	32	70.7	-47.0	7.0						7.4	92.6			STS
	33	73.0	-17.7	3.3						78.4	21.6			STS
	34	62.0	14.7	6.3			23.2	60.4		16.4				Excluded
	35	54.0	44.7	4.7	3.0		97.0							IFG
	36	64.7	66.0	7.0	92.7		7.3							MFG
	43	73.0	-30.7	-8.7							100.0			STS
	44	67.7	-1.7	-10.7						34.0	66.0			STS
	45	56.3	32.7	-7.7			99.1			0.9				IFG
	46	45.0	58.3	-7.3	0.8		99.2							Excluded
	47	21.3	71.7	-5.7	88.1		11.9							MFG

Supplementary Table S2. The post-hoc analyses for the Group x Hemisphere x Region 3-way interaction.

Domain	df	t-statistic	*p*-value	Direction of Effect
**Group-Related Differences**				
Left, MFG	910	-1.175	0.240	ASD > non-ASD
Left, IFG	910	-3.160	**0.002** [a]	**ASD > non-ASD**
Left, PreCG	910	2.468	0.014 [b]	Non-ASD > ASD
Left, PostCG	910	-1.176	0.240	ASD > non-ASD
Left, STS	910	-1.111	0.267	ASD > non-ASD
Left, IPL	876.8	-6.029	**<0.001** [a]	**ASD > non-ASD**
Right, MFG	910	-3.497	**<0.001** [a]	**ASD > non-ASD**
Right, IFG	910	0.887	0.376	Non-ASD > ASD
Right, PreCG	834.1	2.401	0.017 [b]	Non-ASD > ASD
Right, PostCG	910	1.390	0.165	Non-ASD > ASD
Right, STS	910	1.148	0.251	Non-ASD > ASD
Right, IPL	910	3.644	**<0.001** [a]	**Non-ASD > ASD**
**Hemisphere-Related Differences**				
Non-ASD, MFG	440	1.060	0.290	Left > Right
Non-ASD, IFG	440	-5.796	**<0.001** [a]	**Right > Left**
Non-ASD, PreCG	440	0.889	0.374	Left > right
Non-ASD, PostCG	440	-0.123	0.902	Right > Left
Non-ASD, STS	440	3.660	**<0.001** [a]	**Left > Right**
Non-ASD, *IPL*	440	-0.083	0.934	Right > Left
ASD, MFG	470	-2.880	**0.004** [a]	**Right > Left**
ASD, IFG	470	-1.494	0.136	Right > Left
ASD, PreCG	470	1.208	0.228	Left > Right
ASD, PostCG	470	2.672	**0.008** [a]	**Left > Right**
ASD, STS	470	6.482	**<0.001** [a]	**Left > Right**
ASD, *IPL*	470	10.190	**<0.001** [a]	**Left > Right**

a. The *p* values survived the FDR correction
b. The *p* values < 0.05, but not survived the FDR correct

Supplementary Table S3. The post-hoc analyses for the main effect of Condition

**Domain**	**df**	**t-statistic**	***p*-value**	**Direction of Effect**
Watch vs Do	3647	-4.865	**<0.001** [a]	**Do > Watch**
Watch vs Together	3647	-11.055	**<0.001** [a]	**Together > Watch**
Do vs Together	3647	-6.680	**<0.001** [a]	**Together > Do**

a. The *p* values survived the FDR correction
b. The *p* values < 0.05, but not survived the FDR correct
